# Supplementary material for: Infection of 5xFAD mice with a mouse-adapted SARS-CoV-2 does not alter Alzheimer’s disease neuropathology yet induces wide-spread changes in gene expression across diverse cell types
Source: bioRxiv. 2025 Dec 22:2025.12.19.695600. Preprint. [Version 1] doi: 10.64898/2025.12.19.695600 (PMC12767336; doi:10.64898/2025.12.19.695600)

bioRxiv preprint doi: https://doi.org/10.64898/2025.12.19.695600; this version posted December 22, 2025. The copyright holder for this preprint (which was not certified by peer review) is the author/funder, who has granted bioRxiv a license to display the preprint in perpetuity. It is made available under aCC-BY 4.0 International license.

Supplemental Figure 1. (**A**) Quantification of Mac2$^+$ cells and their volumes (**B**) in the cortex, dentate gyrus, and subiculum of MA10-infected WT and 5xFAD mice at day 21 p.i. Immunohistological data were analyzed using two-way ANOVA. Tukey's post-hoc test was employed to examine biologically relevant interactions. Female and male mice are indicated by open or closed circles respectively. Data are represented as mean ±SEM; *** $p \leq 0.001$, **** $p \leq 0.0001$.

Supplemental Figure 2. **MA10 infection and synaptic vesicle density in WT and 5xFAD mice**. Brains of MA10-infected uninfected WT and 5xFAD mice at day 21 p.i. were immunostained with synaptophysin for presynaptic elements (magenta) and PSD-95 for postsynaptic elements (turquoise). (**A**) Representative super-resolution images at 40X objective of cortex, dentate gyrus, and subiculum from infected and uninfected mice are shown. (**B**) Quantification of synaptophysin+ and PSD-95+ colocalized spots per µm3. Immunohistological data were analyzed using two-way ANOVA. Tukey's post-hoc test was employed to examine biologically relevant interactions. Female and male mice are indicated by open or closed circles respectively. Data are represented as mean ±SEM. Scale bar in (A) = 10 µm.

Supplemental Figure 3. Representative images demonstrating cell segmentation in dentate gyrus, choroid plexus, cortex, white matter, striatum and thalamus. Cells were imaged with rRNA (not shown), histone, DAPI, and GFAP markers and segmented automatically.

Supplemental Figure 4. (**A**) Heatmap of top 5 marker genes for all spatial transcriptomics subclusters. (**B**) Proportional distribution of Seurat clusters across experimental groups. Bar plots display relative abundance of each cluster in each group.

Supplemental Figure 5. 46 clusters plotted in XY space in all 6 brains from WT (n=3) and 5xFAD (n=3) mice, both infected and uninfected, at day 21 p.i. (n=3/group).

Supplemental Figure 6. Volcano plots for all spatial transcriptomics subcluster in MA10 infected WT vs Control WT.

Supplemental Figure 7. Volcano plots for all spatial transcriptomics subcluster in MA10 infected 5xFAD vs Control 5xFAD.

Supplemental Figure 8. Investigating gene dysregulation within vascular cells, oligodendrocytes and inhibitory neurons in the presence of amyloid pathology, peripheral MA10 infection, or both concurrently. (**A**) Scatterplot of the average difference of all vascular cells for all significant genes between 5xFAD Control vs. WT Control and WT MA10 vs. WT Control. (**B**) Venn diagram depicting the significant up-and down-regulated genes in vascular cells unique to 5xFAD Control vs. WT Control (left, white) and WT MA10 vs. WT Control (right,

bioRxiv preprint doi: https://doi.org/10.64898/2025.12.19.695600; this version posted December 22, 2025. The copyright holder for this preprint (which was not certified by peer review) is the author/funder, who has granted bioRxiv a license to display the preprint in perpetuity. It is made available under aCC-BY 4.0 International license.

blue), while demonstrating shared dysregulated genes between the two comparisons. (**C**) Scatterplot of the average difference of all vascular cells for all significant genes between 5xFAD Control vs. WT Control. (**D**) Venn diagram depicting the significant up-and down-regulated genes in vascular cells unique to 5xFAD Control vs. WT Control (left, white) and 5xFAD MA10 vs. 5xFAD Control (right, grey), while demonstrating shared dysregulated genes between the two comparisons. (**E**) Three-way Venn diagram of all significantly up- and down-regulated genes within vascular cells between the three comparisons: 5xFAD Control vs. WT Control (white), WT MA10 vs. WT Control (blue), and 5xFAD MA10 vs. 5xFAD Control (grey). (**F**) Scatterplot of the average difference of all oligodendrocytes for all 50 significant genes (padj < 0.05) between 5xFAD Control vs. WT Control (x-axis, only amyloid pathology) and WT MA10 vs. Control WT (y-axis, only MA10 infection) comparisons. Arrows indicate direction of dysregulation for each comparison. Directly correlated genes (blue) occur in the same direction for both comparisons (i.e., both up-regulated or both down-regulated), while inversely correlated genes (orange) occur in opposite directions for each comparison. Linear regression line demonstrates the relationship between the two comparisons. (**G**) Venn diagram depicting the significant up- and down-regulated genes unique to the 5xFAD Control vs. WT Control (left, white) and WT MA10 vs. WT Control (right, blue), while demonstrating up- and down-regulated genes commonly shared across the two comparisons (middle). (**H**) Scatterplot of the average difference of all oligodendrocytes for all significant genes between 5xFAD Control vs. WT Control, now with 5xFAD MA10 vs. 5xFAD Control (y-axis, both amyloid and MA10 infection). Red text demonstrates genes up-regulated in the same direction in the first scatterplot, but down-regulated in the MA10 5xFAD vs. Control 5xFAD comparison. (**I**) Venn diagram depicting the significant up- and down-regulated genes unique to 5xFAD Control vs. WT Control and 5xFAD MA10 vs. 5xFAD Control (right, purple), while demonstrating up- and down-regulated genes commonly shared between the two comparisons. (**J**) Three-way Venn diagram of all significantly up- and down-regulated genes within oligodendrocytes between the three comparisons: 5xFAD Control vs. WT Control (white), WT MA10 vs. WT Control (blue), and 5xFAD MA10 vs. 5xFAD Control (grey). Genes are considered significantly correlated if the log2 Fold Change magnitude is greater than 0.3.(**K**) Scatterplot of the average difference of all inhibitory neurons for all significant genes between 5xFAD Control vs. WT Control and WT MA10 vs. WT Control. (**L**) Venn diagram depicting the significant up- and down- regulated genes in inhibitory neurons unique to 5xFAD Control vs. WT Control (left, white) and WT MA10 vs. WT Control (right, blue), while demonstrating shared dysregulated genes between the two comparisons. (**M**) Scatterplot of the average difference of all inhibitory neurons for all significant genes between 5xFAD Control vs. WT Control. (**N**) Venn diagram depicting the significant up- and down-regulated genes in inhibitory neurons unique to 5xFAD Control vs. WT Control (left, white) and 5xFAD MA10 vs. 5xFAD Control (right, grey), while demonstrating shared dysregulated genes between the two comparisons. (**O**) Three-way Venn diagram of all significantly up- and down-regulated genes within inhibitory neurons between the three comparisons: 5xFAD Control vs. WT Control (white), WT MA10 vs. WT Control (blue), and 5xFAD MA10 vs. 5xFAD Control (grey).

bioRxiv preprint doi: https://doi.org/10.64898/2025.12.19.695600; this version posted December 22, 2025. The copyright holder for this preprint (which was not certified by peer review) is the author/funder, who has granted bioRxiv a license to display the preprint in perpetuity. It is made available under aCC-BY 4.0 International license.

Supplemental Figure 1

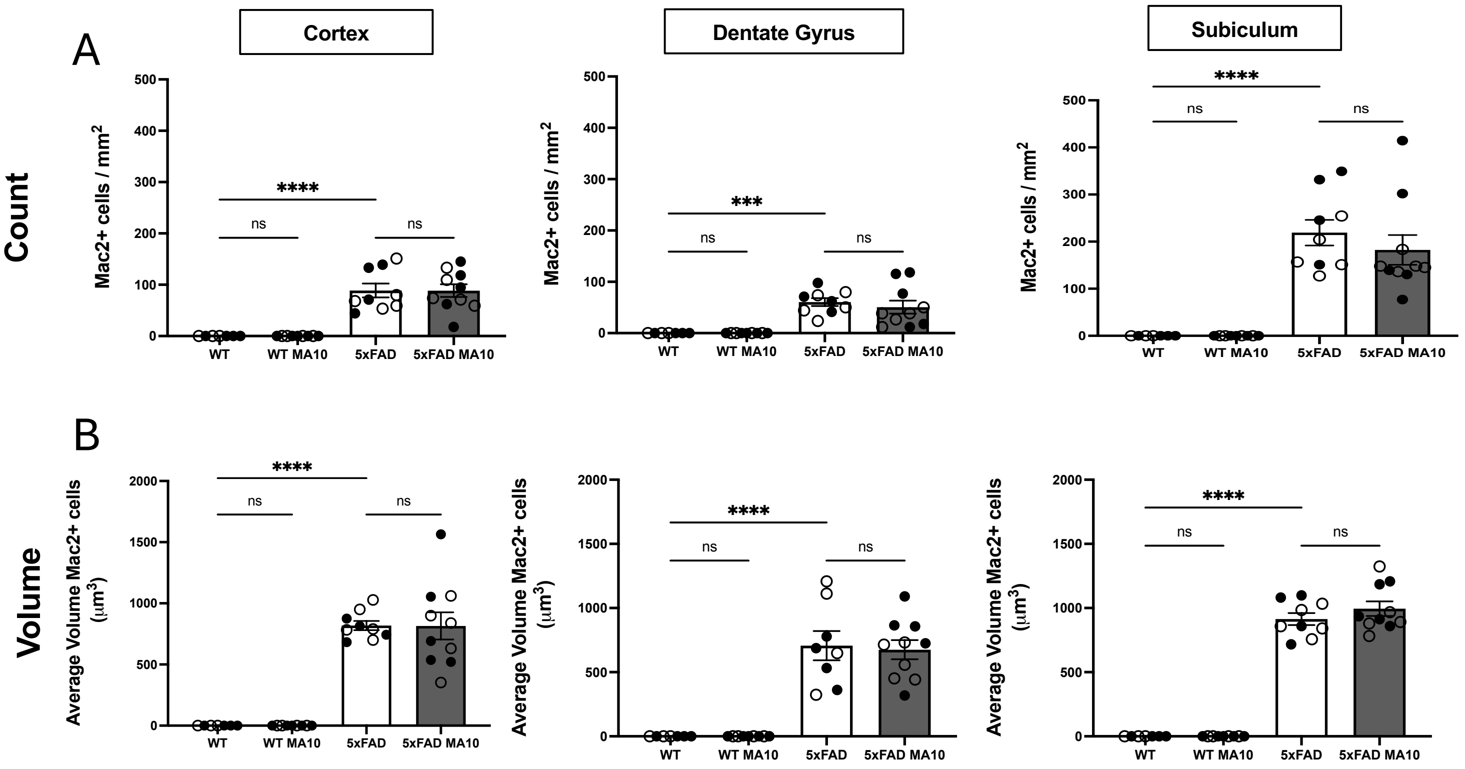

bioRxiv preprint doi: https://doi.org/10.64898/2025.12.19.695600; this version posted December 22, 2025. The copyright holder for this preprint (which was not certified by peer review) is the author/funder, who has granted bioRxiv a license to display the preprint in perpetuity. It is made available under aCC-BY 4.0 International license.

Supplemental Figure 2

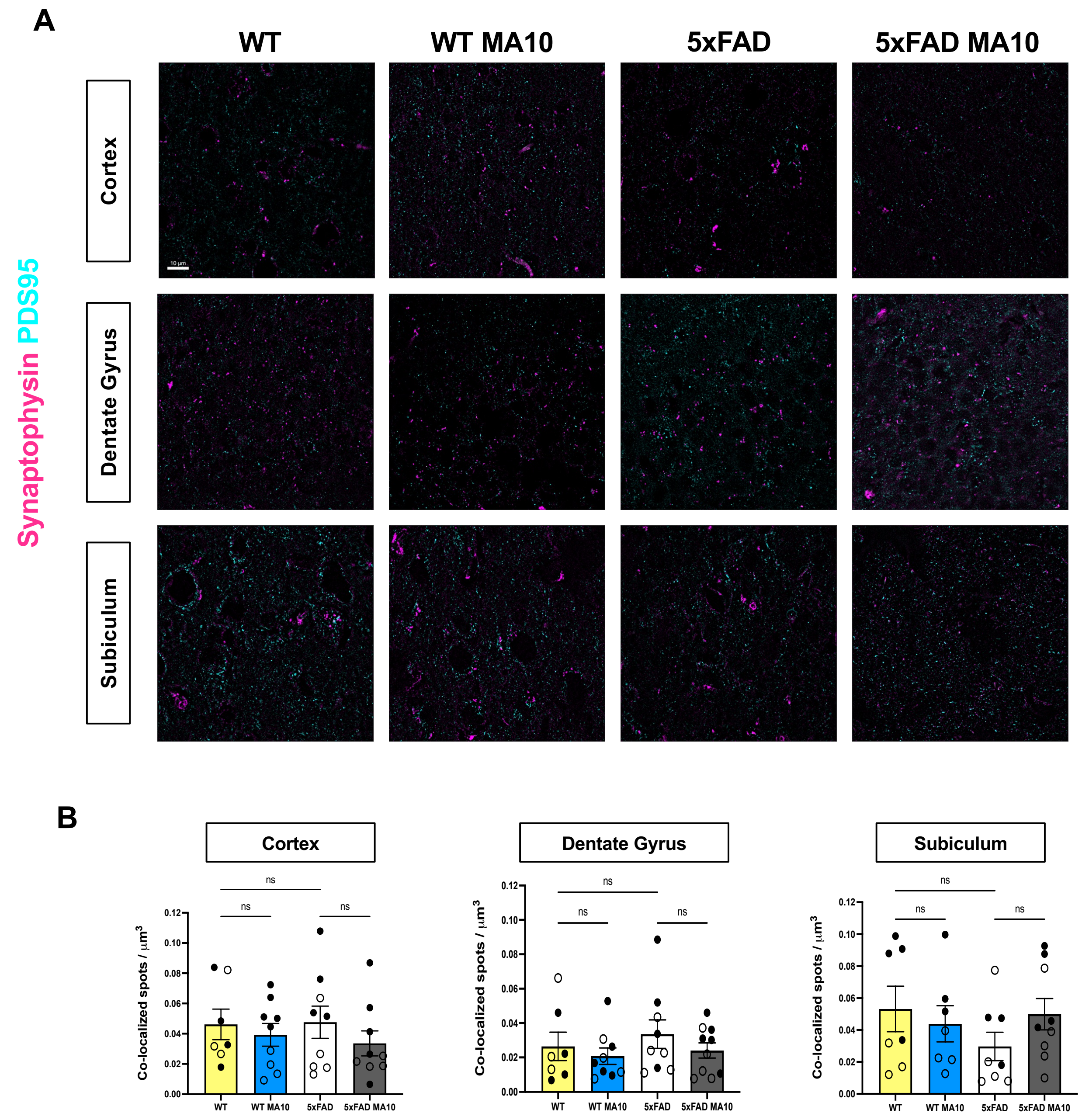

bioRxiv preprint doi: https://doi.org/10.64898/2025.12.19.695600; this version posted December 22, 2025. The copyright holder for this preprint (which was not certified by peer review) is the author/funder, who has granted bioRxiv a license to display the preprint in perpetuity. It is made available under aCC-BY 4.0 International license.

Supplemental Figure 3

Cell segmentation examples

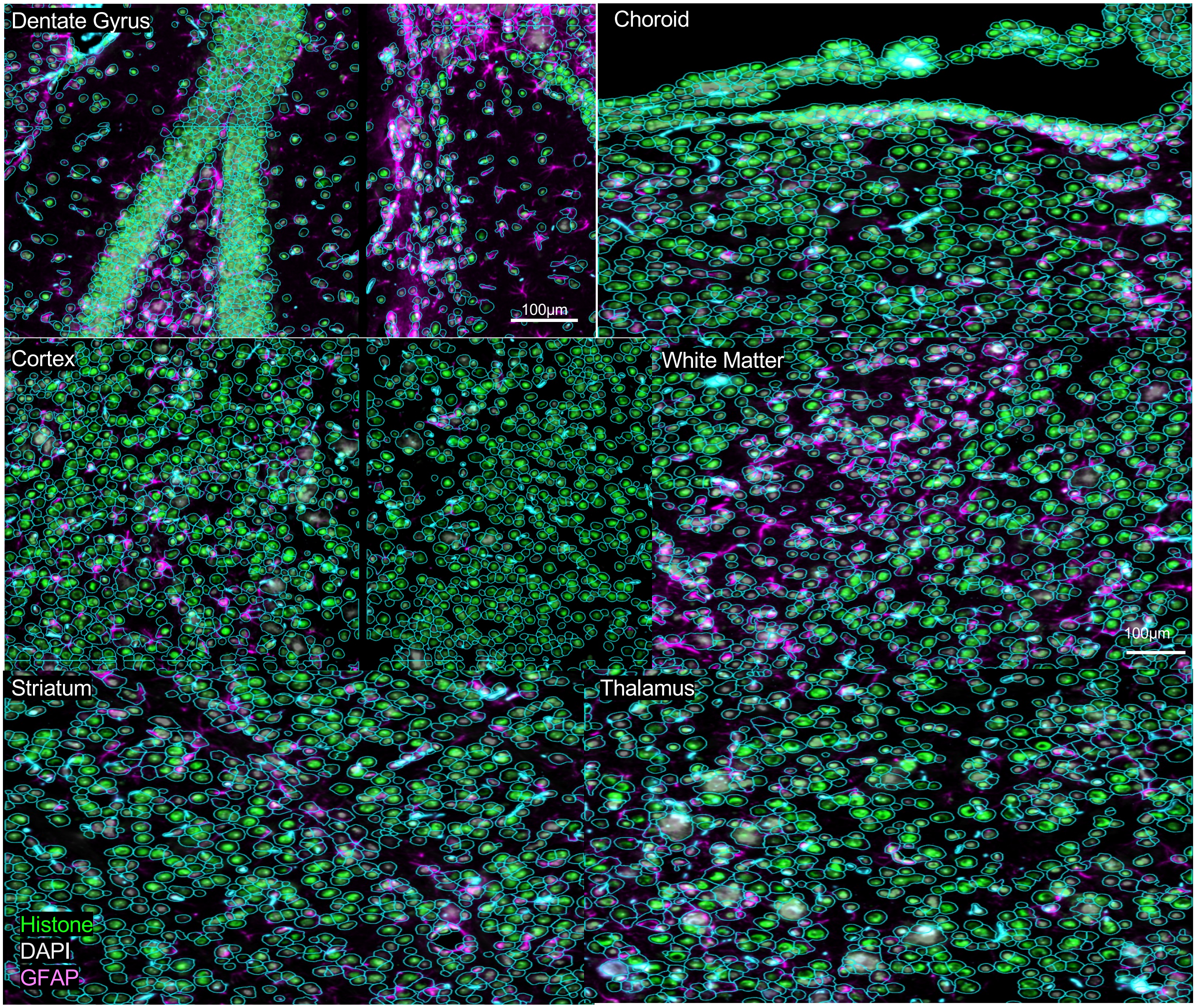

bioRxiv preprint doi: https://doi.org/10.64898/2025.12.19.695600; this version posted December 22, 2025. The copyright holder for this preprint (which was not certified by peer review) is the author/funder, who has granted bioRxiv a license to display the preprint in perpetuity. It is made available under aCC-BY 4.0 International license.

Supplemental Figure 4

A. Top 5 marker genes by cluster

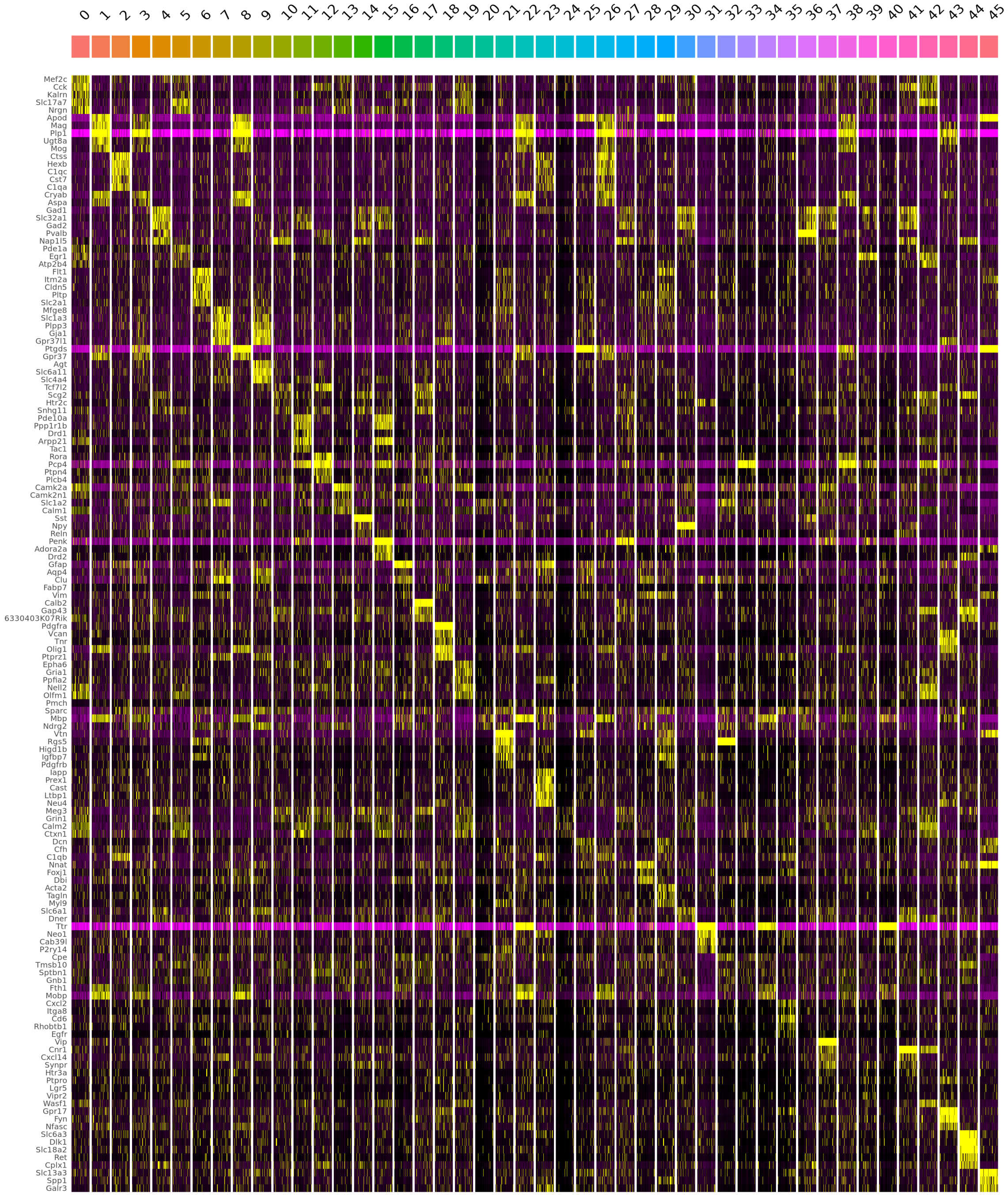

B.

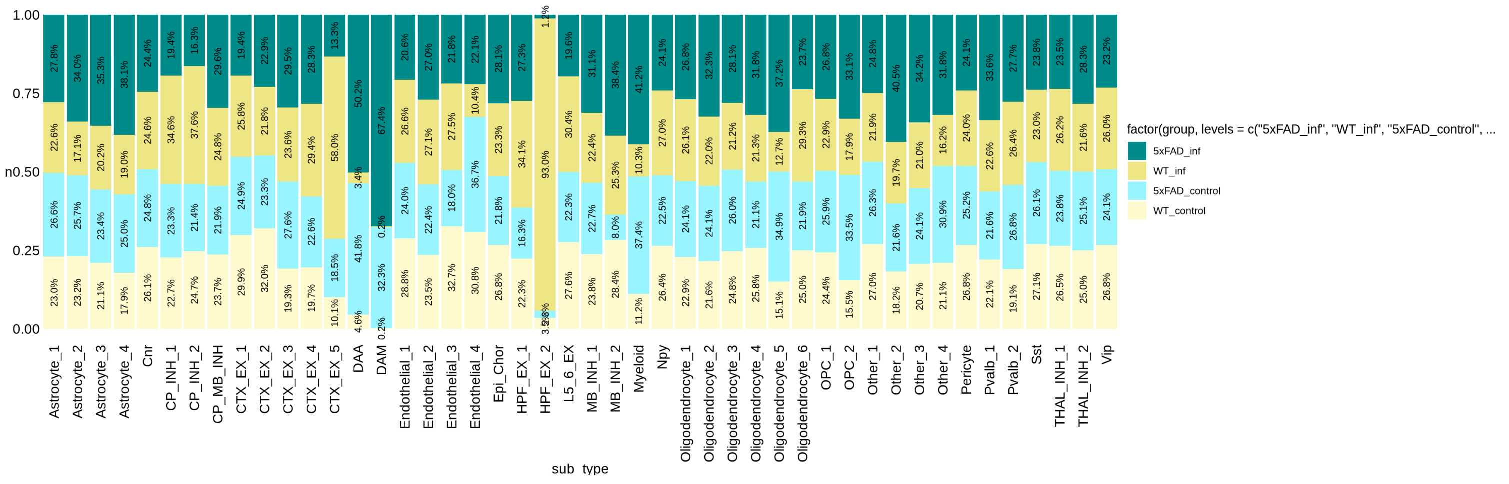

bioRxiv preprint doi: https://doi.org/10.64898/2025.12.19.695600; this version posted December 22, 2025. The copyright holder for this preprint (which was not certified by peer review) is the author/funder, who has granted bioRxiv a license to display the preprint in perpetuity. It is made available under aCC-BY 4.0 International license.

Supplemental Figure 5

Cell clusters in XY space, all brains

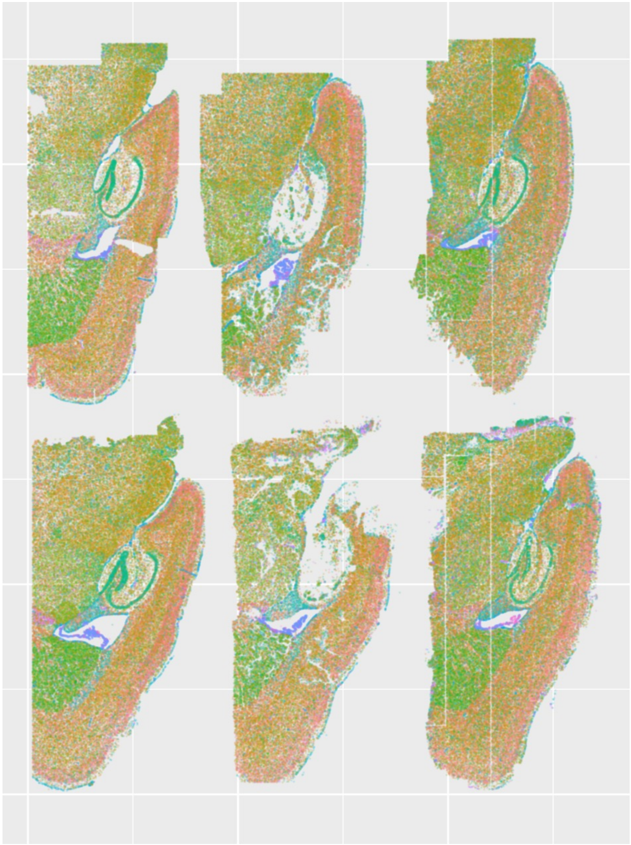

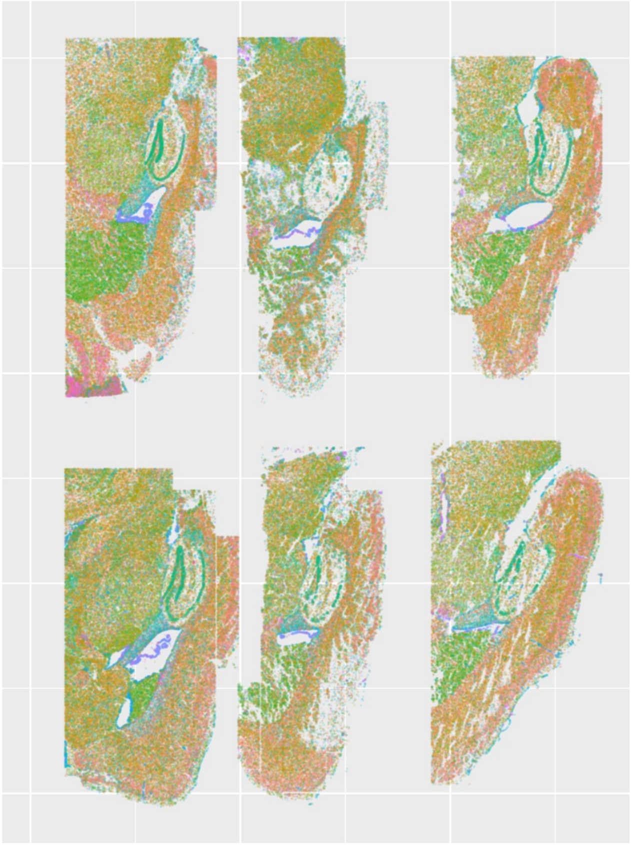

Cluster	Cell type	Sub-type
0	Exitatory_Neuron	CTX_EX_1
1	Olygodendrocyte	Olygodendrocyte_1
2	Myeloid	Myeloid
3	Olygodendrocyte	Olygodendrocyte_2
4	Inhibitory_Neuron	Pvalb_1
5	Exitatory_Neuron	L5_6_EX
6	Vascular	Endothelial_1
7	Astrocyte	Astrocyte_1
8	Olygodendrocyte	Olygodendrocyte_3
9	Astrocyte	Astrocyte_2
10	Inhibitory_Neuron	MB_INH_1
11	Inhibitory_Neuron	CP_INH_1
12	Inhibitory_Neuron	THAL_INH_1
13	Exitatory_Neuron	CTX_EX_2
14	Inhibitory_Neuron	Sst
15	Inhibitory_Neuron	CP_INH_2
16	Astrocyte	DAA
17	Inhibitory_Neuron	THAL_INH_2
18	OPC	OPC_1
19	Exitatory_Neuron	HPF_EX_1
20	Astrocyte	Astrocyte_3
21	Vascular	Pericyte
22	Olygodendrocyte	Olygodendrocyte_4
23	Myeloid	DAM
24	Exitatory_Neuron	CTX_EX_3
25	Other	Other_1
26	Olygodendrocyte	Olygodendrocyte_5
27	Inhibitory_Neuron	CP_MB_INH
28	Astrocyte	Astrocyte_4
29	Vascular	Endothelial_2
30	Inhibitory_Neuron	Npy
31	Epithelial	Epi_Chor
32	Vascular	Endothelial_3
33	Exitatory_Neuron	CTX_EX_4
34	Other	Other_2
35	Vascular	Endothelial_4
36	Inhibitory_Neuron	Pvalb_2
37	Inhibitory_Neuron	Vip
38	Olygodendrocyte	Olygodendrocyte_6
39	Exitatory_Neuron	CTX_EX_5
40	Other	Other_3
41	Inhibitory_Neuron	Cnr
42	Exitatory_Neuron	HPF_EX_2
43	OPC	OPC_2
44	Inhibitory_Neuron	MB_INH_2
45	Other	Other_4

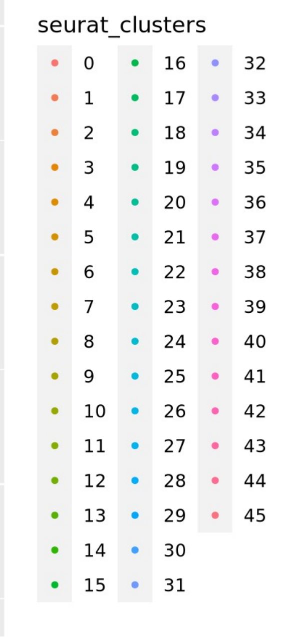

bioRxiv preprint doi: https://doi.org/10.64898/2025.12.19.695600; this version posted December 22, 2025. The copyright holder for this preprint (which was not certified by peer review) is the author/funder, who has granted bioRxiv a license to display the preprint in perpetuity. It is made available under aCC-BY 4.0 International license.

Supplemental Fig 6

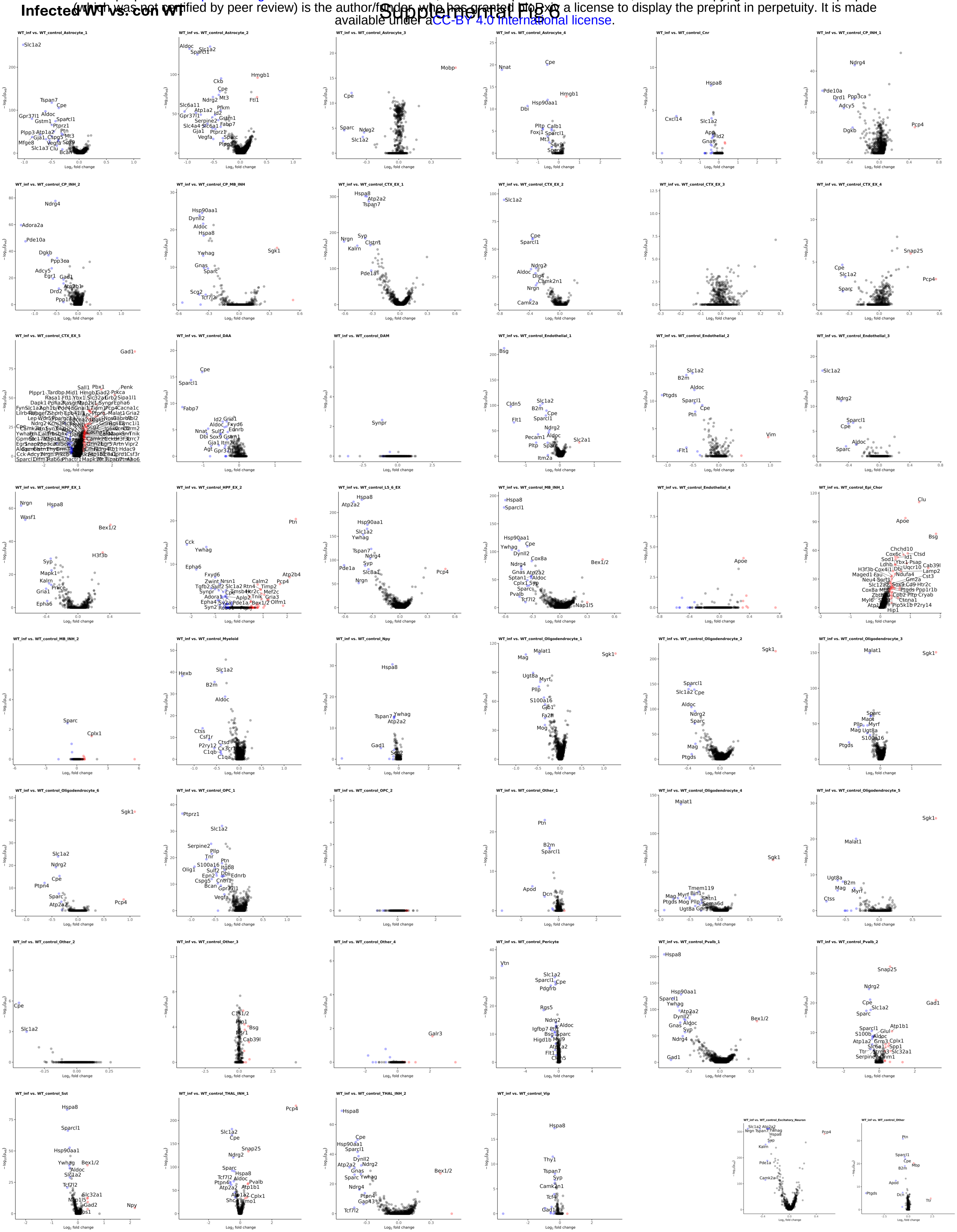

bioRxiv preprint doi: https://doi.org/10.64898/2025.12.19.695600; this version posted December 22, 2025. The copyright holder for this preprint (which was not certified by peer review) is the author/funder, who has granted bioRxiv a license to display the preprint in perpetuity. It is made available under aCC-BY 4.0 International license.

Supplementary Figure 7

Infected 5xFAD vs. con 5xFAD

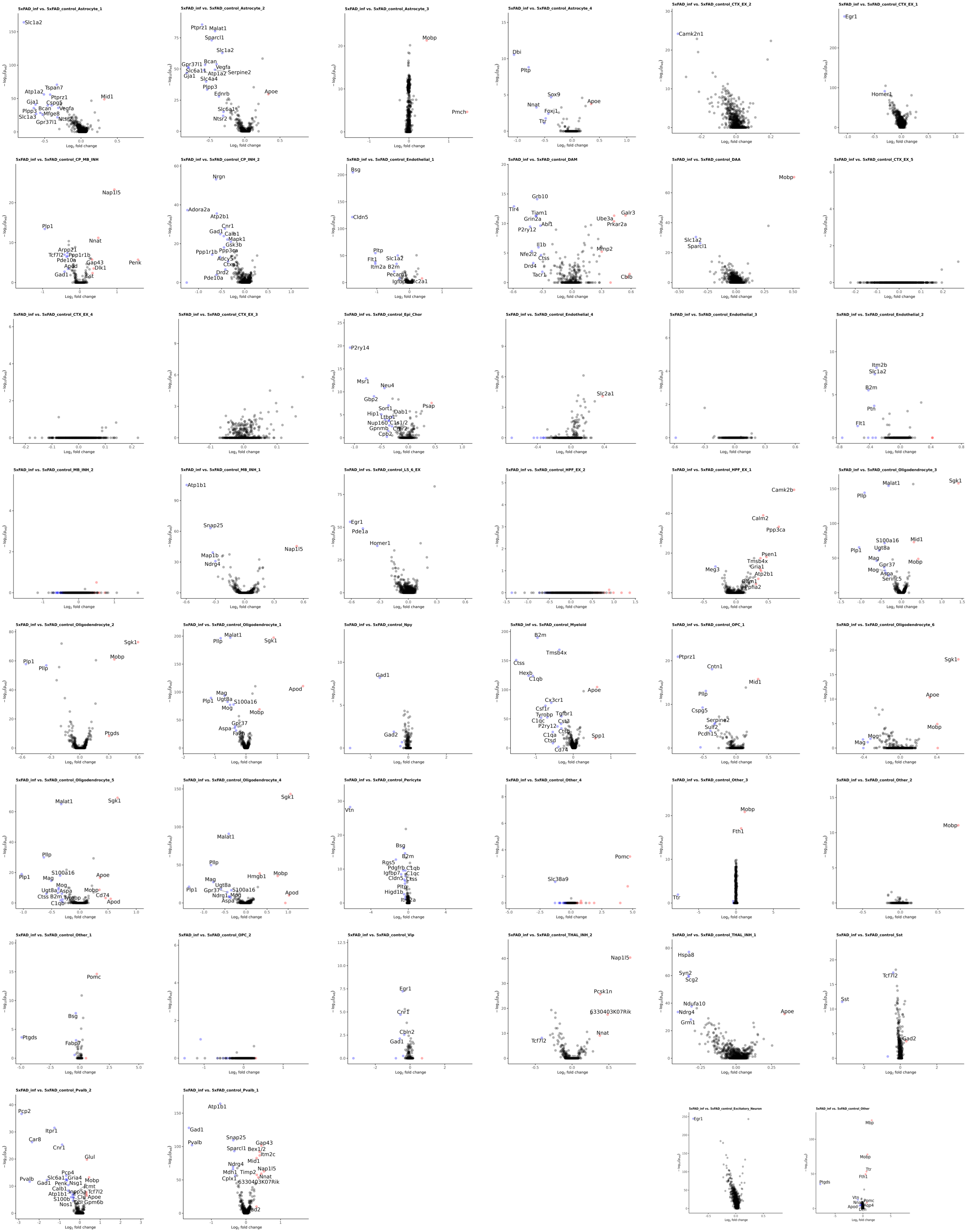

bioRxiv preprint doi: https://doi.org/10.64898/2025.12.19.695600; this version posted December 22, 2025. The copyright holder for this preprint (which was not certified by peer review) is the author/funder, who has granted bioRxiv a license to display the preprint in perpetuity. It is made available under aCC-BY 4.0 International license.

Supplemental Figure 8

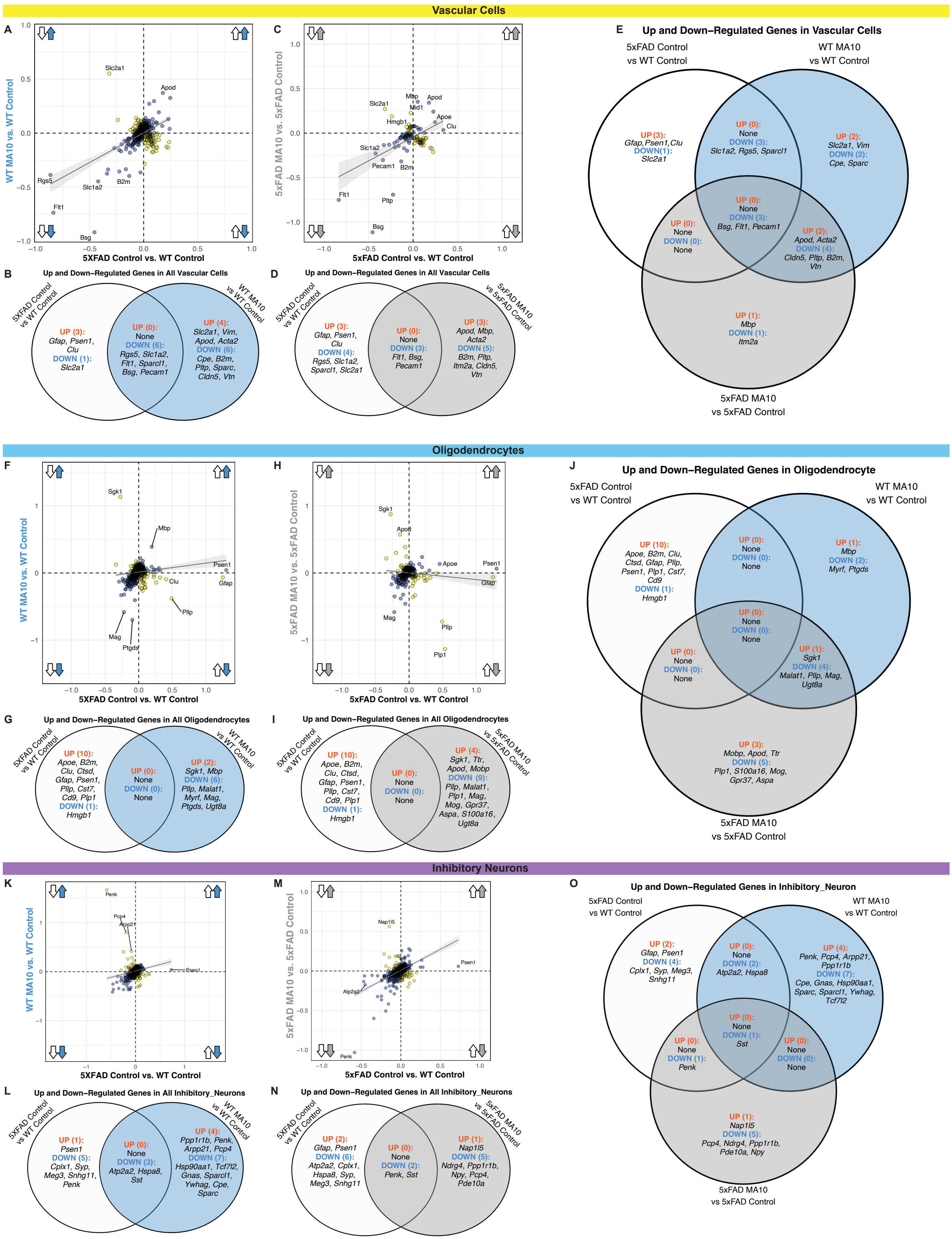

Supplement: Supplement 1 [file NIHPP2025.12.19.695600v1-supplement-1.pdf]
